# Protein force spectroscopy using magnetic tweezers: Slow and steady wins the race?

**DOI:** 10.1016/j.bpj.2025.12.028

**Published:** 2025-12-24

**Authors:** Stefanie D. Pritzl, Jan Lipfert

**Affiliations:** 1 Department of Physics and Debye Institute for Nanomaterials Science, Utrecht University, Princetonplein 1, 3584 CC Utrecht, the Netherlands; 2 Department of Biochemistry and Gruss-Lipper Biophotonics Center, Albert Einstein College of Medicine, Bronx, New York; 3 Institute for Physics, University of Augsburg, Universitätsstrasse 1, 86159 Augsburg, Germany

## Abstract

Mechanical forces are central to biological function across scales, from whole organisms to individual molecules. At the cellular and subcellular levels, force generation, sensing, and mechanotransduction shape diverse processes including gene expression, morphogenesis, and disease progression. Single-molecule force spectroscopy provides critical insights into these mechanics, with magnetic tweezers (MTs) emerging as a versatile tool with unique advantages. MTs operate across physiologically relevant forces (∼0.01–100 pN) and enable stable, long-duration, and multiplexed measurements without photodamage, making them ideally suited to investigate proteins under near-native conditions. This review highlights the evolution of MT-based protein mechanics, spanning early cell microrheology to recent single-molecule studies. We focus on key developments and applications, including investigations of cytoskeletal, membrane, and motor proteins, force-sensitive cell adhesion complexes, mechanoresponsive ion channels, and virus-host interactions. Furthermore, we discuss the integration of MTs with fluorescence readouts and emerging in vivo applications, underscoring the expanding role of MTs in decoding the molecular basis of mechanobiology.

## Significance

Mechanical forces play a central role in regulating protein function across cellular processes, yet tools to precisely probe protein mechanics under physiological conditions remain limited. Magnetic tweezers overcome key challenges by enabling long-term, stable, low-force measurements at the single-molecule level. This review highlights how magnetic tweezers have advanced our understanding of protein folding, force sensing, and mechanotransduction, spanning cytoskeletal components, membrane receptors, and in vivo systems. Their versatility and compatibility with fluorescence detection position magnetic tweezers as a cornerstone technique for uncovering how mechanical cues shape protein behavior and cellular function.

## Introduction

Mechanical forces are critical for biological processes at scales ranging from organisms to organs and tissues down to single macromolecules. Examples include the action of molecular motors, shear flow, and tensile stretch and compression in tissues. Forces affect and regulate, for example, cell morphology, proliferation, and differentiation (1,2). Recent work has shown how gene expression depends on the mechanics of the nucleus (3) and has highlighted the role of mechanical stability in how host-pathogen interactions (4) and how cell mechanics (5) can affect the onset and evolution of diseases such as cancer (6), asthma (7), or hemostatic disorders (8). It is therefore crucial to understand the biomechanical processes and their specific roles in a cellular context, in particular force generation and sensing, and the transduction to biochemical signals and metabolic action. Proteins are key players that allow cells to sense, generate, and translate forces, which renders them important research targets to develop a quantitative, bottom-up understanding of how forces and protein nanomechanics affect and regulate living systems (9).

Proteins in biological systems often experience mechanical forces as part of molecular assemblies or when they are anchored to larger structures, for example, membranes or the cytoskeleton, that can transmit external loads. Since most biological activity occurs near ambient temperature, the characteristic thermal energy is approximately *k*_B_*T* ≈ 4 pN⋅nm. At this energy scale, thermal fluctuations are substantial and continuously perturb molecular components. To carry out directed motion or perform mechanical tasks in this noisy environment, molecular machines such as motor proteins must generate forces that exceed this thermal background. Considering that their typical step size (*d*) is in the nanometer range, the minimal force required to bias motion or resist fluctuations can be estimated as *F* ∼ *k*_*B*_*T*/*d*, which falls in the piconewton range. This simple scaling argument explains why many biological force-generating processes operate within a narrow range of ∼1–10 pN. Measured forces typically fall between 1 and 100 pN, defining the key operational window for single-molecule force spectroscopy (10).

**Figure 1:**
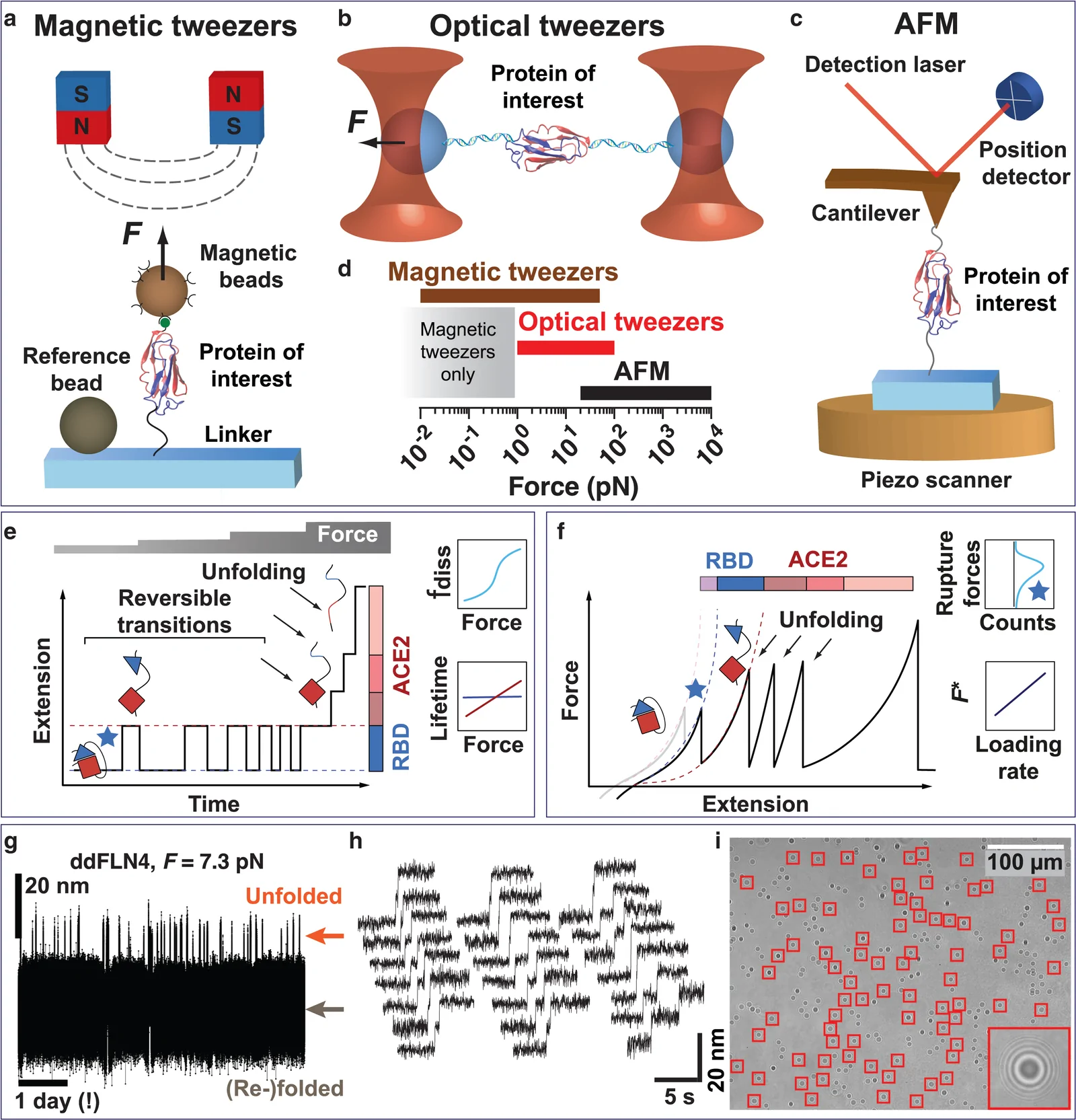
Comparison of single-molecule force spectroscopy techniques and capabilities of magnetic tweezers. (*a*–*d*) Common single-molecule force spectroscopy techniques and typical force ranges accessible to them. Schematics of magnetic tweezers (*a*), optical tweezers (*b*), and AFM-based (*c*) single-molecule measurements of a protein of interest. The protein shown is the mechano-protein ddFLN4. (*d*) Force ranges accessible to the techniques are shown schematically in (*a*)–(*c*). Partially adapted from (11). (*e* and *f*) Schematic representations of the types of data that are obtained in constant force measurements (such as in magnetic tweezers, *e*) and in constant loading rate measurements (in particular in AFM-based force spectroscopy, *e*). Example schematic data are shown for a SARS-CoV-2 tethered ligand construct (12) (Fig. 3). Constant force measurements record molecular extension vs. time at different levels of force and can monitor repeated reversible transitions or irreversible unfolding events. Data are usually summarized as the fraction dissociated or unfolded and as lifetimes vs. force. Constant loading rate measurements record force vs. extension. In the resulting saw-tooth pattern, each peak corresponds to one dissociation or unfolding event. Data are summarized as the distribution of rupture forces and finally as the most probable rupture force vs. loading rate. Reprinted (adapted) with permission from *Proc. Natl. Acad. Sci. USA* 2022, *119* (14), e2114397119 (12). Copyright 2022, the Author(s). Published by PNAS and distributed under Creative Commons License 4.0 (CC BY-NC-ND). (*g*–*i*) Capabilities of magnetic tweezers measurements. (*g*) Week-long, constant force protein unfolding and refolding measurement of ddFLN4 demonstrates excellent stability (13). (*h*) Extension-time traces of ddFLN4 unfolding acquired in parallel within a single field of view at an applied force of 21 pN, revealing characteristic three-state unfolding behavior. Note: traces are offset in time and extension axes to enhance distinguishability. Reprinted (adapted) with permission from *Proc. Natl. Acad. Sci. USA* 2019, *116* (38), 18,798–18807 (13). Copyright 2019, the Author(s). Published by PNAS and distributed under Creative Commons License 4.0 (CC BY-NC-ND). (*i*) Representative field of view from a magnetic tweezers experiment, with red squares indicating 78 beads tracked simultaneously. Inset: magnified diffraction pattern of a single bead used for precise position tracking. Reprinted (adapted) with permission from *Biophys. J.* 2024, 123, 3964–3976 (14). Copyright 2024, the Authors. Published by Elsevier Inc. on behalf of Biophysical Society.

Several single-molecule force spectroscopy (SMFS) techniques have been developed to probe the mechanical response of proteins to external forces, with atomic force microscopy (AFM), optical tweezers (OT), and magnetic tweezers (MTs) being the most widely employed (Fig. 1). AFM is particularly well suited for applying and measuring high force, exceeding 10–20 pN and extending into the nanonewton range (Fig. 1 *c* and *d*) (15,16,17). However, its large and relatively stiff cantilever renders it less effective for resolving low forces (<10 pN) (15). OTs, on the other hand, operate optimally within an intermediate force range (1–100 pN) and offer exceptional spatiotemporal resolution (Fig. 1 *b* and *d*) (18,19). Despite these advantages, OTs suffer from inherent limitations, including low throughput and the risk of photodamage or local heating caused by the trapping lasers (20). In addition to these established methods, approaches such as flow stretch, centrifuge force microscopy (CFM), and acoustic force spectroscopy (AFS) have emerged. CFM enables massively parallel measurements by exploiting centrifugal forces applied to tethered beads (21,22,23), whereas AFS employs acoustic standing waves to simultaneously exert calibrated forces on large ensembles of molecules (24,25). Although attractive for their throughput and versatility, these methods have thus far been applied mainly to nucleic acids and ligand-receptor systems, and their utility in protein mechanobiology remains to be demonstrated.

MTs overcome many limitations of other SMFS techniques and have emerged as a versatile and powerful platform, initially mostly for nucleic acids (26,27,28), but recently and increasingly also for studying protein mechanics. In MT assays, the molecule of interest is tethered between a functionalized surface and a micrometer-sized superparamagnetic bead (Fig. 1 *a* and *d*). By adjusting the position and orientation of external magnets, well-controlled forces that range from ∼0.01 pN to over 100 pN (and torques, from ∼1 pN·nm to thousands of pN·nm), can be applied to the bead, enabling precise manipulation and observation of molecular systems across the full spectrum of physiologically relevant forces (17,11,29,30,31,32,33,34,35). Unlike techniques relying on optical or thermal input, MTs do not introduce sample heating or photodamage, which makes them uniquely suited for extremely long-duration experiments and compatible with studies in living cells (36,37,38). Another distinguishing feature of MTs is their ability to operate in passive constant-force mode: once the magnets are positioned, the applied force remains stable without the need for active feedback control (Fig. 1 *e*) (30,39). In contrast, OT and in particular AFM-based force spectroscopy measurements typically operate in the regime of constant loading rate (Fig. 1 *f*). Constant force measurements allow for robust probing of force-dependent molecular transitions over diverse time scales, extending from submilliseconds (40,41) to weeks (3,42) (Fig. 1 *g*). In addition, recent advances in camera technology, real-time tracking algorithms, and GPU-accelerated data processing now enable simultaneous tracking of hundreds to thousands of individual molecules (Fig. 1 *h* and *i*) (3,43,44,45). This multiplexing capability is critical for detecting rare events, analyzing irreversible reactions, and obtaining statistically meaningful insights from heterogeneous systems (46).

Another key strength of MTs is their compatibility with fluorescence-based detection (47,48,49), including Förster resonance energy transfer (FRET), allowing mechanical manipulation to be correlated with structural or biochemical readouts. This capability has expanded the utility of MTs well beyond nucleic acid mechanics, positioning them as a powerful tool in modern biophysical research. Their applications now span force-dependent conformational changes, protein-protein interactions, and enzymatic processes.

**Figure 2:**
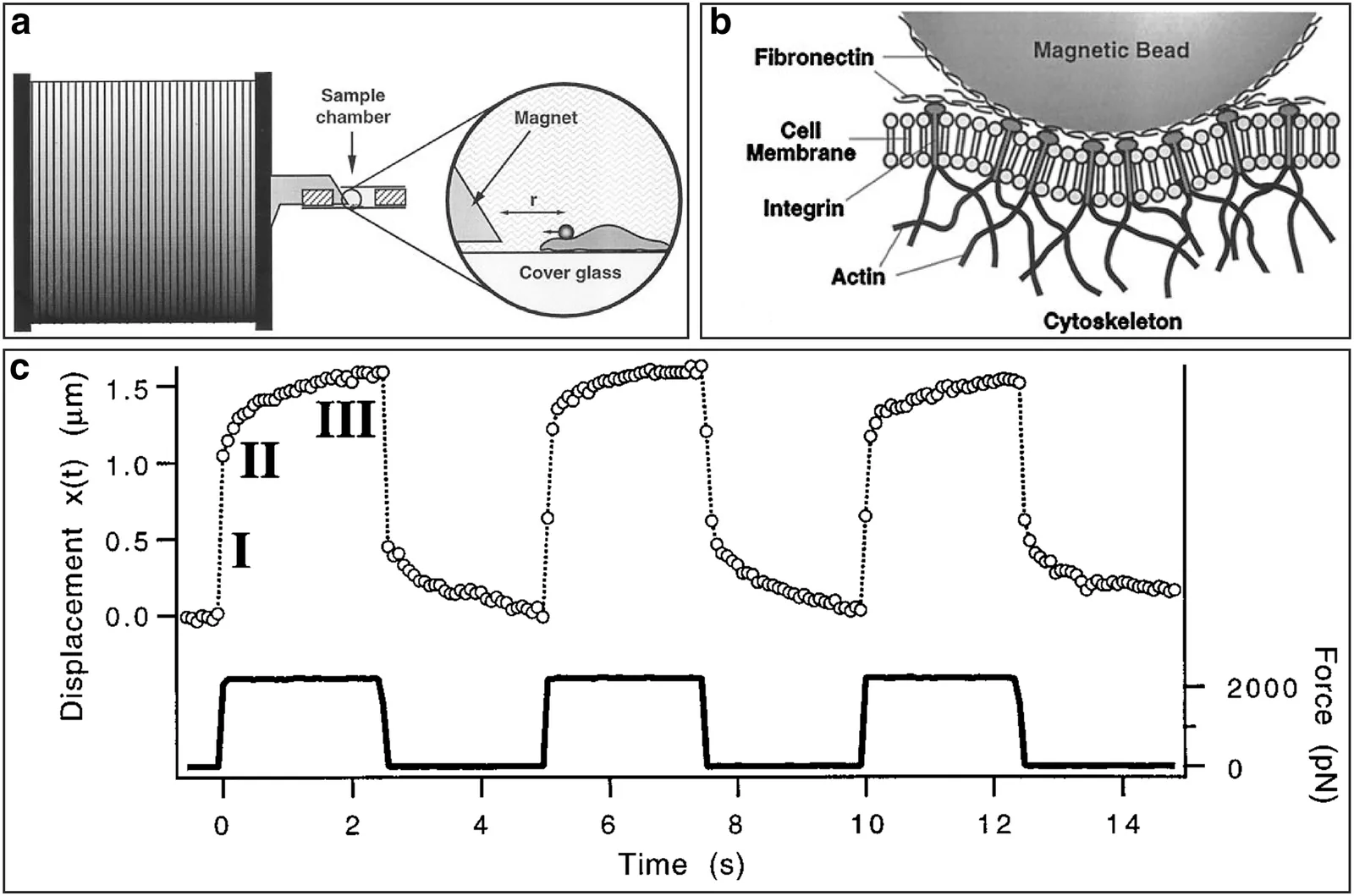
Early pioneering MT work from the Sackmann lab. (*a*) Central component of the magnetic bead rheometer. A soft iron-core electromagnet (1200-turn coil) delivers forces up to 10,000 pN on a 4.5-μm bead, with the pole tip positioned 10–100 μm from the sample. The assembly is mounted on a microscope stage for precise control. (*b*) Schematic illustration of a fibronectin-coated bead linked to the cell cytoskeleton through integrin-mediated adhesion. (*c*) Representative creep and relaxation response of a 4.5-μm bead bound to the membrane of a 3T3 fibroblast via fibronectin-integrin interactions. Although this setup probes collective, cellular-scale mechanics rather than single molecules, it effectively interrogates mechanoprotein responses within the adhesion network, thereby establishing the conceptual foundation for later single-molecule MT studies. Force pulses of 2000 pN were applied for 2.5 s. Reprinted (adapted) with permission from *Biophys. J.* 1998, 75, 2038–2049 (52). Copyright 1998, The Biophysical Society. Published by Elsevier Inc.

The versatility of MTs has driven their application from early cellular mechanics assays to the current frontier of protein force spectroscopy. Initial MT-like experiments, such as those pioneered by Crick and Hughes (50) in the 1950s and later refined by Bausch, Sackmann, and colleagues (51,52) in the 1990s, applied forces to magnetic beads attached to cells to extract viscoelastic properties including elasticity, viscosity, and strain relaxation (Fig. 2). Around the same period, the now-standard MT configuration emerged, in which individual biomolecules or complexes are tethered between a flow-cell surface and a superparamagnetic bead, with applied forces and torques modulated through external magnets.

Different MT configurations are routinely used depending on the intended measurement. In conventional MTs, vertical forces are applied to study processes such as DNA (over-)stretching and supercoiling, protein unfolding, and the force dependence of protein-protein interactions (17,11,39). In freely orbiting MTs (FOMTs), beads are allowed to rotate, enabling high-resolution studies of DNA twisting and nucleic acid supercoiling (30,31,32,36,37,38,40,53,54). Magnetic torque tweezers, in contrast, restrict bead rotation and permit precise measurement of torque by analyzing angular deviations under controlled magnetic constraints (41).

Altogether, MTs now represent one of the most refined and flexible techniques for probing biomolecular mechanics at the single-molecule level. They have been extensively applied to study the mechanical properties and interactions of nucleic acids with proteins and enzymes like topoisomerases, cold shock proteins, nucleosomes, and telomeric proteins as well as single-stranded DNA-binding proteins like GapR and structural maintenance of chromosome protein complexes (45,46,54,55,56,57,58,59,60,61,62,63,64,65,66,67,68). Novel configurations such as junctured DNA-tweezers (69), at which a double-stranded DNA (dsDNA) serves as scaffold to enchase the molecule of interest, continue to expand the MT toolkit for exploring protein dynamics and interactions.

In this article, we highlight early pioneering work in the MT field, including applications of magnetic tweezers to cells from the Sackmann lab and review MT-based developments and reports in the field of protein mechanics to date focusing on protein interactions, recent progress toward fluorescence-force spectroscopy, and in vivo applications, complementing previous reviews (10,70,71,72,73,74,75,76,77).

## Main

SMFS experiments (Fig. 1 *a*–*d*) typically yield two principal readouts: force-extension curves (Fig. 1 *f*) and extension-time traces (Fig. 1 *e*). Force-extension measurements reveal unfolding transitions, contour length changes, and elastic responses as the applied force is varied. Extension-time traces, recorded under constant force, capture stochastic folding/unfolding events, bond lifetimes, and conformational switching (Fig. 1 *e*). MTs naturally operate in the force-clamp mode, which is particularly powerful for direct determination of protein kinetics under well-defined load. Depending on the assay design, MTs can probe single-domain unfolding and refolding, tethered ligand interactions, protein-protein complexes, or enzymatic activity. These modern applications build on early pioneering work, where MTs were first adapted for microrheology of living cells, probing the viscoelastic response of cytoskeletal protein networks under mechanical load.

### Early pioneering work in the MT field

In the late 1990s, Sackmann and colleagues developed the use of MTs for microrheology of live cells with high precision (Fig. 2) (51,52). They attached 4- to 4.5-μm paramagnetic beads to adherent fibroblasts via integrin receptors and applied stepwise tangential forces ranging from 500 pN to ∼3000 pN (Fig. 2 *a* and *b*). These force pulses, sustained for approximately 1 s, elicited characteristic three-phase creep responses: an initial elastic deflection, a stress-relaxation phase, and a subsequent viscous flow (Fig. 2 *c*). By analyzing these responses, which are modeled as combinations of dashpot and Voigt elements, they quantified the cell’s effective elastic constant, viscosity, and relaxation time. They also mapped the spatial extent of the bead-induced deformation by tracking nearby nonmagnetic tracer beads, revealing that the resulting displacement field decays to baseline within roughly 7 μm from the bead. The local shear elastic modulus was measured between 20 and 735 Pa, whereas viscosity reached approximately 210 Pa·s, reflecting the complex, viscoelastic behavior of the cytoskeleton. Importantly, these rheological properties are largely determined by the underlying cytoskeletal protein network, including actin filaments, intermediate filaments, and associated crosslinkers, which dominate the cell’s mechanical response. These studies also established partial penetration of mechanical perturbations into the cytoplasm and demonstrated highly localized mechanical compartments within the cell. Beyond their immediate contribution to probing cell mechanics, this seminal work in “magnetic bead microrheometry” provided an experimental foundation for later MT studies, bridging cellular-scale microrheology with the subsequent development of single-protein MT assays.

### Methodological consideration for single-molecule magnetic tweezers measurements

MT experiments for the manipulation and measurement of individual molecules were first pioneered for nucleic acids (26,78). Subsequently, the method has been extended to individual proteins. In both cases, several methodological challenges need to be addressed to enable stable and high-resolution measurements.

#### Tethering strategies

To achieve high-resolution measurements, mechanical stability of the tethered construct is essential. A critical factor is the anchoring strategy linking the bead, the molecule of interest, and the substrate (10). Commonly used streptavidin-biotin linkages, though robust, exhibit direction-dependent behavior and a broad distribution of lifetimes under load (79,80). Comparative studies of streptavidin variants with different valencies and defined tether geometries have identified specific configurations that significantly extend tether lifetimes, enabling ultrastable SMFS experiments (79). Alternative strategies include Traptavidin- or AviTag-biotin ligation, covalent coupling using epoxy- or carboxylic-acid-coated beads, and engineered tags such as SpyTag or HaloTag for surface attachment (10,81).

#### Instrumentation and MT calibration

In addition to biochemical tethering, physical design parameters influence experimental performance. The applied force in MTs scales with the gradient of the magnetic field and with the cube of the bead radius $F\;\propto \;R_{bead}^{3}$, making bead size a critical factor in force calibration (42,70). Moreover, off-center tethering leads to asymmetric force transmission and can induce bead rotation due to anisotropic magnetization along the field axis (82). This introduces additional torques from both the magnetic field and the restoring tension of the tether, which must be accounted for in high-precision measurements. Tether length further affects system stiffness and response times, necessitating careful optimization for reliable calibration and resolution.

Technological refinements have addressed these challenges, improving the calibration of applied forces, optimizing imaging speeds, and expanding dynamic force control (14,42,43,44,47,48,49,53,83,84). Permanent magnets, which are widely used in MTs, provide robust and uniform force application across broad fields of view (42) but are limited in the speed of force modulation. To enhance performance, pole pieces can be used to locally shape magnetic fields, increasing achievable forces at the expense of spatial uniformity. Electromagnetic configurations such as those using magnetic tape heads, enable rapid modulation of force at frequencies above 10 kHz (85,86), making it possible to resolve fast-folding intermediates such as molten globule states. Additionally, hybrid MT setups now combine the benefits of high force sensitivity with extended dynamic ranges (≥500 pN), further expanding their utility across molecular systems (87,88). Recent protocols and technical advances now also provide standardized guidance for MT instrumentation, calibration, and multiplexing strategies, thereby facilitating reproducibility and broader adoption of MTs across laboratories (89).

### Applications of MTs in protein force spectroscopy

MTs have proven highly effective in probing mechanical properties of proteins at the single-molecule level, particularly under constant, low-force conditions. They have been used to compare structurally distinct proteins in terms of their folding and unfolding forces and kinetics, force-dependent domain stabilities, associated contour length changes, and unfolding-refolding patterns (90,91,92,93). MTs have also enabled detailed studies of protein-protein interactions, addressing both stability and dynamics, as well as of environmental influences such as ion-binding (94) and ionic strength (95) down to subpiconewton forces. Importantly, although many of these proteins have been previously examined using AFM or OT, MTs have offered unique insights by facilitating stable, long-duration measurements under well-defined and constant forces, especially in the low-force regime (<10 pN), where thermal fluctuations dominate and other methods are less reliable.

#### Probing muscle and cytoskeletal proteins at constant forces

One important application of MTs is the study of muscle and cytoskeletal proteins under constant mechanical load. Muscle cells rely on the coordinated activity of contractile proteins to generate force for skeletal and cardiac function (96). Loss of this functionality, often due to genetic mutations, underlies conditions such as muscular dystrophy and cardiomyopathy (97,98). A central protein in this process is titin, the largest known human protein, whose immunoglobulin (Ig) domains can refold under tension and perform mechanical work exceeding that of myosin motors (99). MT studies of titin’s I27 domain revealed nonmonotonic unfolding at forces below 100 pN, with a minimum unfolding rate near 22 pN (100). Folding and unfolding rates equilibrate around 5.4 pN (101), and stepwise refolding below 10 pN generates up to 105 zJ of contractile energy (102). These results, made possible by the stable force-clamp and long observation times in MTs, extend AFM and OT findings by capturing slow, subtle transitions. Similar behavior in cardiac titin near 5 pN aligns with its role in buffering mechanical load during heartbeats (103). MTs also revealed stochastic unfolding and refolding of dystrophin at forces below 25 pN, supporting its role as a molecular shock absorber, insights not easily obtained via ramp-based techniques (104,105). These studies highlight the advantage of MTs over AFM, which probes fast unfolding events at high loading rates, by enabling direct observation of slow refolding steps and long-lived equilibrium states under constant load.

In the cytoskeletal context, MTs have probed proteins such as actin and its crosslinkers including talin (106), α-actinin (107), and filamin A (FLNa) (39,108). Cyclic force measurements have suggested that talin acts as a mechanical band-pass filter, responding to specific force frequencies (109). MTs also uncovered that the Rho-activated formin mDia1 accelerates actin polymerization under low force (0.5–10 pN), but only when filaments are torsionally unconstrained (110,111). Lastly, MT and denaturation studies of titin’s I83 domain revealed differences in mechanical response across techniques, underscoring the need for method-aware analysis (112). These findings highlight the unique capacity of MTs to study force-dependent protein mechanics under near-physiological conditions, in particular to apply sustained (sub-)piconewton forces over long timescales, uncovering frequency-dependent filtering and torsion-sensitive polymerization that OT or AFM could not reliably capture.

#### Membrane proteins

Cellular membranes host a diverse array of proteins that enable communication, transport, and signal transduction between cellular compartments and their surroundings (113), making them crucial targets for pharmaceutical intervention. Although AFM has effectively revealed unfolding dynamics and energy landscapes of membrane proteins (114), MTs again offer the advantage of operating at physiologically relevant forces, even down to subpiconewton levels (115,116). This makes MTs particularly well suited to probe protein folding dynamics and conformational stability under gentle, constant force conditions.

Studies using MTs have provided valuable insights into membrane-associated proteins. For instance, Guo et al. (117) employed MTs to determine the force-dependent folding and unfolding dynamics of the GB1 subunit of G-protein over a force range of 3–160 pN, which has not been accessible by AFM or OT. A nonmonotonic force-dependent unfolding rate of GB1 was found, revealing two main energy barriers and a hidden intermediate state. Similarly, experiments on the tight-junction protein ZO-1 showed that small tensions, on the order of 1–4 pN, are sufficient to maintain its stretched conformation, whereas forces between 5 and 20 pN disrupt intraprotein interactions (118). The spectrin repeat SR73 of nesprin, a nuclear envelope mechanosensor, was shown to refold stepwise under constant force after complete unfolding, highlighting its potential role in transmitting mechanical stimuli from the cytoskeleton to the nucleus (119).

MTs have also allowed protein dynamics to be studied in lipid environments that mimic cellular membranes. This includes work on *E. coli* rhomboid protease GlpG, which was found to unfold cooperatively under forces above 25 pN, exhibiting hysteresis and refolding only at forces below 5 pN (120,121,122). Additional applications have extended to voltage-gated and mechanosensitive ion channels. For example, MT experiments revealed force-sensitive domains within the Piezo1 channel that regulate its activation and inactivation under forces below 10 pN (123). Similarly, in the BK channel, 6 pN stimuli produced significant hyperpolarization within minutes (124). The transmembrane receptor NOTCH1 (125) was shown to trigger signaling only under forces of 1.4 pN or in the presence of ligand and slightly higher forces, and TREK1 channel (126) activity was similarly enhanced by mechanical input.

MTs have recently also provided insights into the membrane fusion machinery. Soluble N-ethylmaleimide-sensitive factor attachment protein receptor (SNARE) proteins, which mediate vesicle fusion (127), were shown to unzip at approximately 34 pN and rezip via a mechanically distinct intermediate around 11 pN, suggesting mechanical hysteresis and directionality (128). Furthermore, the folding dynamics of all-α proteins were probed using acyl-coenzyme A binding protein, where MTs revealed a switch in folding pathways depending on whether forces exceeded or fell below 6 pN (129). Such examples demonstrate that MTs complement and can outperform AFM, which excels at high-force rupture, and OT, which can suffer from heating and drift, by providing stable low-force clamps that uncover hidden intermediates and subtle conformational equilibria in membrane proteins.

Furthermore, MTs have been used to study adhesion G-protein-coupled receptors (aGPCRs), a subclass of the GPCR superfamily, revealing how mechanical forces modulate receptor conformation and signaling. aGPCRs are membrane proteins with extended extracellular domains that undergo autoproteolysis to form noncovalently associated fragments. Force-mediated dissociation of these fragments is believed to activate G-protein signaling mechanisms (130,131,132). MT experiments showed that physiologically relevant forces (1–10 pN) can unfold the GAIN domain and trigger dissociation of the N- and C-terminal fragments, especially in latrophilin-1 and GPR56, on a seconds-to-minutes timescale, supporting a model in which a force-sensitive equilibrium between compact and extended GAIN states precedes dissociation (133). Further single-molecule MT studies across different aGPCR subfamilies (ADGRG1/GPR56, ADGRL1/LAT-1, and noncleavable ADGRB3/BAI3) revealed that forces of just a few piconewtons induce GAIN domain unfolding and NTF:Stachel dissociation (134). Even during cell migration, sufficient extracellular linkage enables this dissociation. In a mechanosensory context, the GPCR LPHN2 (ADGRL2) in vestibular hair cells was shown to transduce force into electrical response: it senses shear forces to increase TMC1 channel open probability, triggering glutamate release for equilibrioception, underscoring the physiological relevance of mechanosensitive aGPCR signaling (135). These aGPCR studies underscore the particular strength of MTs in resolving slow dissociation dynamics under a few piconewtons of force, which are difficult to capture with AFM’s rapid pulling or the lower stability of OTs in this regime.

#### Mimicking force-activation of von Willebrand factor in hemostasis

**Figure 3:**
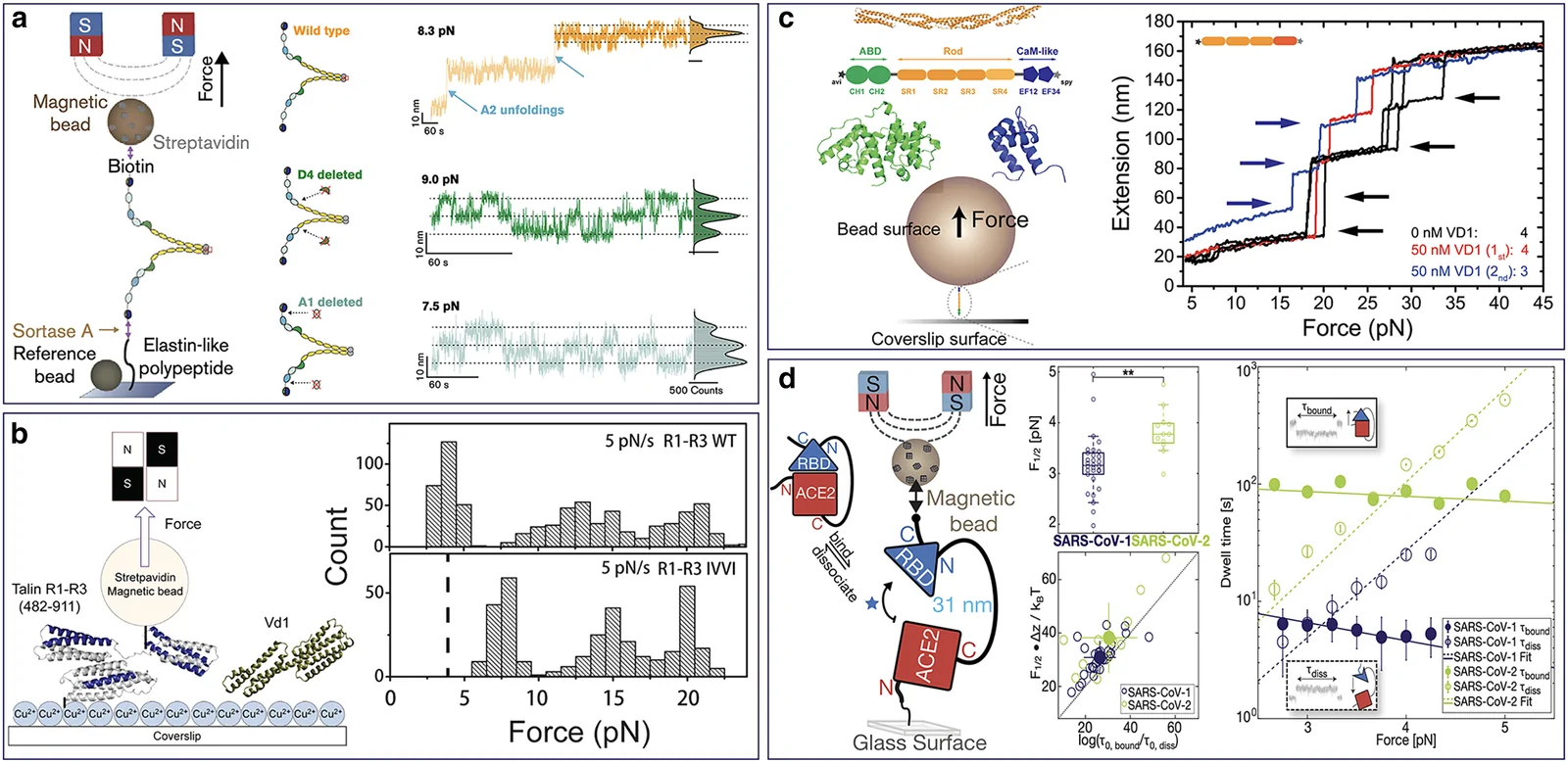
Protein force spectroscopy in MTs. (*a*) Schematic of a vWF dimer in magnetic tweezers. vWF is tethered to the flow cell via an elastin-like polypeptide (ELP) linker and coupled to a paramagnetic bead using a biotin–streptavidin bond. Reference beads correct for drift, and forces are applied via two permanent magnets. Right: three-state transitions in vWF dimers are observed regardless of A2, D4, or A1 domain presence. Extension-time traces at constant forces (7.5–9.0 pN) show distinct transitions, confirmed by histograms fitted with three-term Gaussians, indicating domain-independent conformational switching. Reprinted (adapted) with permission from *Blood Adv.* 2022, *6* (17), 5198–5209 (142). Copyright 2022, American Society of Hematology (ASH). (*b*) Left: schematic of the experimental setup. A talin rod construct is tethered between a cover glass and a paramagnetic bead via NTA–His and streptavidin–biotin linkages. Right: force is applied with permanent magnets, and constructs are stretched with or without vinculin VD1. The unfolding force distributions of wild-type talin R1–R3 (*top*) and the IVVI mutant (*bottom*) at a 5 pN s^−1^ loading rate are ≤23 pN. Reprinted (adapted) with permission from *Sci. Rep.* 2014, 4, 4610 (143). Copyright 2014, Springer Nature under Creative Commons License 3.0 (CC BY-NC-SA). (*c*) Left: schematic structure of α-actinin-1 with its functional domains, i.e., ABD, rod, and CaM-like. Right: force-extension responses of the rod construct before (*black*) and after (*red, blue*) addition of VD1, measured at a loading rate of ∼1 pN s^−1^. Reprinted (adapted) with permission from *Cell Rep.* 2017, *21* (10), 2714–2723 (107). Copyright 2017, Elsevier. (*d*) Left: Schematic (not to scale) of the magnetic tweezers tethered ligand assay. The construct links ACE2 (*red square*) and RBD (*blue triangle*) via a flexible polypeptide (85 or 115 aa), anchored covalently to the surface and to a magnetic bead via biotin-streptavidin. Stretching forces are applied using permanent magnets. Middle top: SARS-CoV-2 RBD:ACE2 bonds show significantly higher force stability than SARS-CoV-1 (*p* = 0.00124, ^∗∗^; two-tailed *t*-test). Each point represents F_1/2_ from an individual molecule; boxes show medians and interquartile ranges. Middle bottom: free energy differences from equilibrium (F_1/2_⋅Δx) and dynamic (log(τ_0,bound_/τ_0,diss_) data are consistent and indicate a more stable SARS-CoV-2 interface. Data from 29 (SARS-CoV-1) and 12 (SARS-CoV-2) molecules. Right: bound (*solid*) and dissociated (*open*) dwell times for SARS-CoV-1 (*blue*) and SARS-CoV-2 (*green*). Means ± SD from exponential fits (*n* = 29, 12). Dashed/solid lines: bound/dissociated fits; insets: example traces. Reprinted (adapted) with permission from *Proc. Natl. Acad. Sci. USA* 2022, *119* (14), e2114397119 (12). Copyright 2022, the Author(s). Published by PNAS and distributed under Creative Commons License 4.0 (CC BY-NC-ND).

The glycoprotein von Willebrand factor (vWF) is a key player in primary hemostasis. vWF travels in the bloodstream as a long multimer of vWF dimers, which are activated by shear forces at sites of vascular injuries (136). Although force-induced conformational changes of vWF trigger platelet and collagen adhesion, reduced activity or mutations can lead to bleeding disorders, thrombosis, and infarction (137,138,139). Multiple pH-dependent interactions within dimeric subunits of vWF define its conformation and mechanics (140), and force sensing is strongly dependent on intermonomer interactions (141). Using MTs, our group recently studied the impact of individual domains on conformational changes and multimerization of vWF and their force dependence (Fig. 3 *a*). MT measurements revealed pH-dependent conformational changes in the N-terminal (D’D3) domain, which are believed to be critical for vWF multimerization (142), and Ca^2+^ binding was found to stabilize the A2 domain, which unfolds under physiological forces (13). Finally, MTs were able to induce and monitor transitions in the C domain stem, which “unzips” and “rezips” at forces of ≈1 pN (13). These vWF experiments highlight the unparalleled stability of MTs for long-duration, subpiconewton to tens-of-piconewton force application, which was essential to dissect gradual, domain-specific transitions that AFM or OT could not resolve with equal precision.

### Force-dependent protein interactions and adhesion

Protein-protein interactions are structurally and functionally diverse, varying in composition, affinity, and whether they form transient or stable associations (144). Chaperones, for instance, play essential roles in biosynthetic protein folding and adhesion (145). MT studies have shown that the chaperone trigger factor modulates the folding dynamics of protein L over a force range of 4–10 pN, enhancing folding probability and accelerating refolding kinetics under load (145). Similarly, unfoldase chaperones reduce talin’s unfolding force from ∼11 pN to ∼6 pN, whereas foldase chaperones increase it to ∼15 pN, thereby reshaping talin’s folding energy landscape and stabilizing adhesion complexes (146). Recent MT studies also revealed that p47, a cofactor of the AAA+ ATPase p97, enhances talin’s mechanical stability independently of ATPase activity (147). Though not a classical chaperone, p47 acts like a foldase by stabilizing proteins under force. It shifts talin’s folding midpoint from 8.4 to 16.6 pN and promotes a compact conformation, boosting mechanical work output. These results position p47 as a force-sensitive modulator of protein energy landscapes in mechanoregulated proteostasis. In tissues, protein interactions can affect cell adhesion to the extracellular matrix (ECM) or to neighboring cells through the formation of cell-ECM and/or cell-cell junctions. The precise transmission of mechanical force from cell to ECM and cell to cell is pivotal for cell homeostasis and function.

#### Cell-ECM junctions and interactions

A key player in signaling pathways that result in cell-ECM interaction via the activation of integrins, e.g., via vinculin, is talin (148,149). Talin acts as structural scaffold for linking the contractile cytoskeleton and integrin/fibronectin adhesion (150). It is involved in, e.g., cell migration and spreading by sustaining stretching and vinculin binding cycles that are governed by contractile forces during retral movement of actin filaments (151). Thereby, ECM forces are transformed into biochemical signals to regulate and stabilize the initial adhesion junctions and to transduce them through the integrins (152,153,154,155). For vinculin, MT studies revealed that its binding by talin is strongly force dependent. It was shown that upon mechanical force and stretching of talin R1–R3, binding of vinculin is enhanced (156) and favored by unfolding talin and exposing the vinculin-binding sites (143). The reorganization of the vinculin-binding site helices was suggested to cause binding events of the unfolded talin polypeptide observed at forces ≤23 pN (Fig. 3 *b*) (143). In addition, the primary mechano-sensing domain in talin was identified as stabilizing mutations of R3 that unfold at 5 pN, which implies a 5 pN threshold for vinculin binding and adhesion progression (156). Furthermore, it was reported that vinculin exhibits dynamic fluctuations between an autoinhibited closed and open conformation, which are stabilized upon binding to the vinculin-binding site in talin (157). These insights into talin-vinculin mechanotransduction were enabled by the stable force clamp provided by MTs, which allowed direct observation of gradual binding thresholds and force-dependent site exposure.

Another important cytoskeletal protein involved in cell migration and cross-linking actin filaments at sites of cell-ECM junctions is α-actinin. Like talin, α-actinin can bind to vinculin and plays a role in maturation of adhesions via force transmission (107,158). It was previously suggested that force is required to activate α-actinin for high-affinity vinculin-binding (159,160). Using MTs, Le et al. (107) showed that application of mechanical force results in unfolding of the SR4 domain of α-actinin and full exposure of the αVBS (vinculin binding site) for high-affinity binding of the vinculin D1 domain (Fig. 3 *c*). In a complementary approach, a tethered-ligand assay was employed with a flexible peptide linker that covalently connects the ligand and receptor between a magnetic bead and a surface, enabling the application of calibrated stretching forces and the direct observation of repeated binding/unbinding cycles within the same molecular construct (161). This configuration allows precise quantification of force-regulated binding kinetics under constant load, revealing that VBS-VD1 (vinculin binding site and vinculin domain 1) complexes remain stable for up to 1000 s at ≤10 pN but dissociate within seconds at 15–25 pN, deviating from Bell’s model predictions and better described by an exponential model with a force-dependent energy barrier based on the structural-elastic property of the complex.

In a comparative MT-based in vitro assay of talin and α-actinin, it was further shown that the two compete for binding β_3_ integrin (fibronectin and vitronectin) but cooperate in binding to β_1_ integrins (collagen) (162). Based on experimental data, the authors suggest a multistep process that enables cells to adjust forces in ECM: in step 1, talin links nascent β_3_-containg adhesions to the actin cytoskeleton. Then (step 2), talin and α-actinin compete to bind β_3_ integrins, before (step 3) adhesion maturation is triggered by force transmission through α-actinin. Last (step 4), after adhesion maturation, α-actinin is recruited in correlation to force generation. Again, MTs provided the long-term stability at moderate forces necessary to dissect this multistep adhesion maturation process, which relies on slow competition and cooperative binding dynamics.

#### Cell-cell junctions and interactions

Cadherins mediate cell-cell adhesion through adherens junctions and play a central role in mechanotransduction. MT studies have shown that applying tension to C-cadherins induces polarized protrusions, cytoskeletal reorganization, and recruitment of plakoglobin to adhesion sites, which are key steps for force-sensitive junction assembly (163). MTs were also used to investigate α-catenin, the main linker between cadherins and actin (164). Forces around 5 pN triggered unfolding of α-catenin and enabled vinculin binding, stabilizing its open state. Additionally, force spectroscopy on cadherin-23 variants revealed that single-point mutations alter intradomain interactions and β-strand dynamics, producing distinct force-adaptation behaviors (165). Variants with stronger internal contacts resisted higher forces and maintained conformational states longer, whereas others showed faster folding but more force-sensitive energy landscapes, linking mechanical properties to genotype-phenotype variation.

#### SNARE-mediated interactions

Neuronal SNARE complexes play a central role in driving membrane fusion, with the energy required for this process generated through the stepwise zippering of SNARE proteins. The transition between the resting state and fusion-competent conformations is tightly regulated by accessory proteins, including complexin (Cpx) (166,167,168). MTs have been used to dissect the molecular details of SNARE-Cpx interactions. In the force range of 12–16 pN, rapid transitions between intermediate conformations of individual neuronal SNARE complexes were detected. These studies also revealed that Cpx stabilizes the assembled SNARE complex while simultaneously preventing full zippering by interfering with the assembly of the SNARE linker domains. This dual behavior suggests that Cpx functions as a finely tuned clamp, regulating the activation threshold of the neuronal SNARE machinery. Here, MTs offer the advantage of finely controlling intermediate force ranges over extended durations, capturing the rapid switching between SNARE conformations in real time and in equilibrium.

#### Antibodies

Antibodies are affinity proteins that can specifically bind invaders with high affinity, act as adaptor molecules, and recruit immune cells. In recent years, various antibody engineering approaches have emerged (169), aiming for the development of efficient and stable therapeutics. In this regard, the low force range accessible by MTs is very useful to determine protein bond dynamics and kinetics, and stability with high accuracy. By applying forces in the range 1.5–12 pN, the interaction between the immunoglobulin IgG and protein A was characterized, revealing a weak and a strong slip bond that were attributed to the interaction of protein A with the constant region (Fc) and heavy chain variable domain (VH) of IgG, respectively (170). Furthermore, the amount of antibody ligands can greatly impact protein domain stability. For protein L, antibody binding was reported to increase mechanical protein stability as a consequence of binding to the low-avidity binding site that acts as a mechanosensor (171).

#### Intrinsically disordered proteins

Intrinsically disordered proteins (IDPs) play key roles in signaling, transcription, and cytoskeletal organization, where structural flexibility enables interaction with multiple partners. Using MTs, the entropic elasticity of an IDP derived from the neurofilament protein tail was measured, revealing a low-force power-law regime consistent with Pincus scaling (172). The extracted Flory exponent increased with denaturant, indicating a shift from ideal to swollen chain behavior. These results demonstrate the potential of force spectroscopy to probe IDP conformations and their environmental responsiveness. These measurements are enabled by the ability of MTs to apply stable subpiconewton forces, which are necessary to capture polymer-like elasticity regimes and scaling behavior that AFM or OT cannot easily resolve.

#### Viral infection

Forces and protein-mediated interactions also play key roles in viral infections and the uptake of viruses in human host cells. Only recently, this has been proven critical for the severe acute respiratory syndrome coronavirus (SARS-CoV). The implementation of a MT-based assay allowed our group to investigate the attachment of the receptor-binding domain (RBD) on the viral Spike protein to the extracellular domain of angiotensin-converting enzyme-2 (ACE2) under mechanical load (Fig. 3 *d*) (12). In this “tethered ligand assay,” the RBD and ACE2 domains are again covalently linked via flexible peptide linkers within the same construct, allowing repeated and well-defined binding/unbinding cycles of a single molecular pair under controlled force in a tethered-ligand assay. This design eliminates complications from diffusive rebinding and enables repeated measurements of the interaction to obtain statistics to quantify how force modulates viral attachment and receptor engagement. The force dependence and kinetics of the RBD:ACE2 bond in equilibrium were measured at forces of 2–5 pN, and a higher mechanical stability, larger binding free energy, and a lower dissociation rate of SARS-CoV-2 than of SARS-CoV-1 were found (Fig. 3 *d*), which likely contribute to the different viral infection patterns and, ultimately, to the spread of the disease. MTs further revealed that although all tested SARS-CoV-2 variants of concern show increased ACE2 affinity, only the Alpha variant also exhibits enhanced force stability, highlighting mechanical resilience as a potential factor in viral fitness (173). This viral interaction work underscores the ability of MTs to characterize equilibrium bond lifetimes and subtle affinity differences in the low-piconewton range, which are central to infection biology but lie outside the optimal working regime of AFM and OT.

### Fluorescence-force spectroscopy: Combining MTs with fluorescence readouts

With the progress of MT instruments to achieve accurate and precise force measurements with subpiconewton resolution, the need for orthogonal information to study more complex protein systems is also increasing. One strategy is the combination of MTs with fluorescence microscopy, which has already been proven suitable to, e.g., implement evanescent nanometry (174) and protein tracking (102,175) by using fluorescent magnetic beads or nanoparticles. Also, single fluorophores have been applied to identify binding events of vinculin to talin rods upon mechanical talin rod unfolding and exposing of previously buried binding sites (176) and to study the replication protein A and its function in DNA replication, repair, and recombination (177). Furthermore, a combination of confocal microscopy and MTs has been used to investigate the link between cellular morphogenetic movements and its coupling to nuclear function in a developmental context revealing, e.g., force-dependent nuclear morphology alterations and morphogenetic defects that impact gene expression (178).

A particularly powerful extension is the combination of MTs with single-molecule Förster resonance energy transfer, which provides molecular-level insight into distances and conformational dynamics. This approach has been used to track enzymatic fluctuations and their effects on activity, as demonstrated by Guo et al. (179,180), and to reveal ATP-dependent SNARE disassembly by NSF at single-molecule resolution as shown by Ryu et al. (181). Building on this, a recent approach combines MTs with total internal reflection fluorescence microscopy to study CRISPR-Cas complexes (182). Fluorescent labeling of the Cascade surveillance complex allows direct observation of DNA binding and release, whereas MTs simultaneously detects DNA unwinding during R-loop formation. This integrated platform also enables tracking of 1D protein diffusion along stretched DNA, significantly expanding the experimental scope for studying a broad range of protein-DNA interactions. Here, the advantage of MTs lies in their ability to apply stable, constant forces in the low-piconewton range while simultaneously recording fluorescence signals, enabling correlated mechanical and conformational readouts that AFM’s surface geometry and the laser-induced perturbations of OT make more challenging.

### Toward in vivo applications

**Figure 4:**
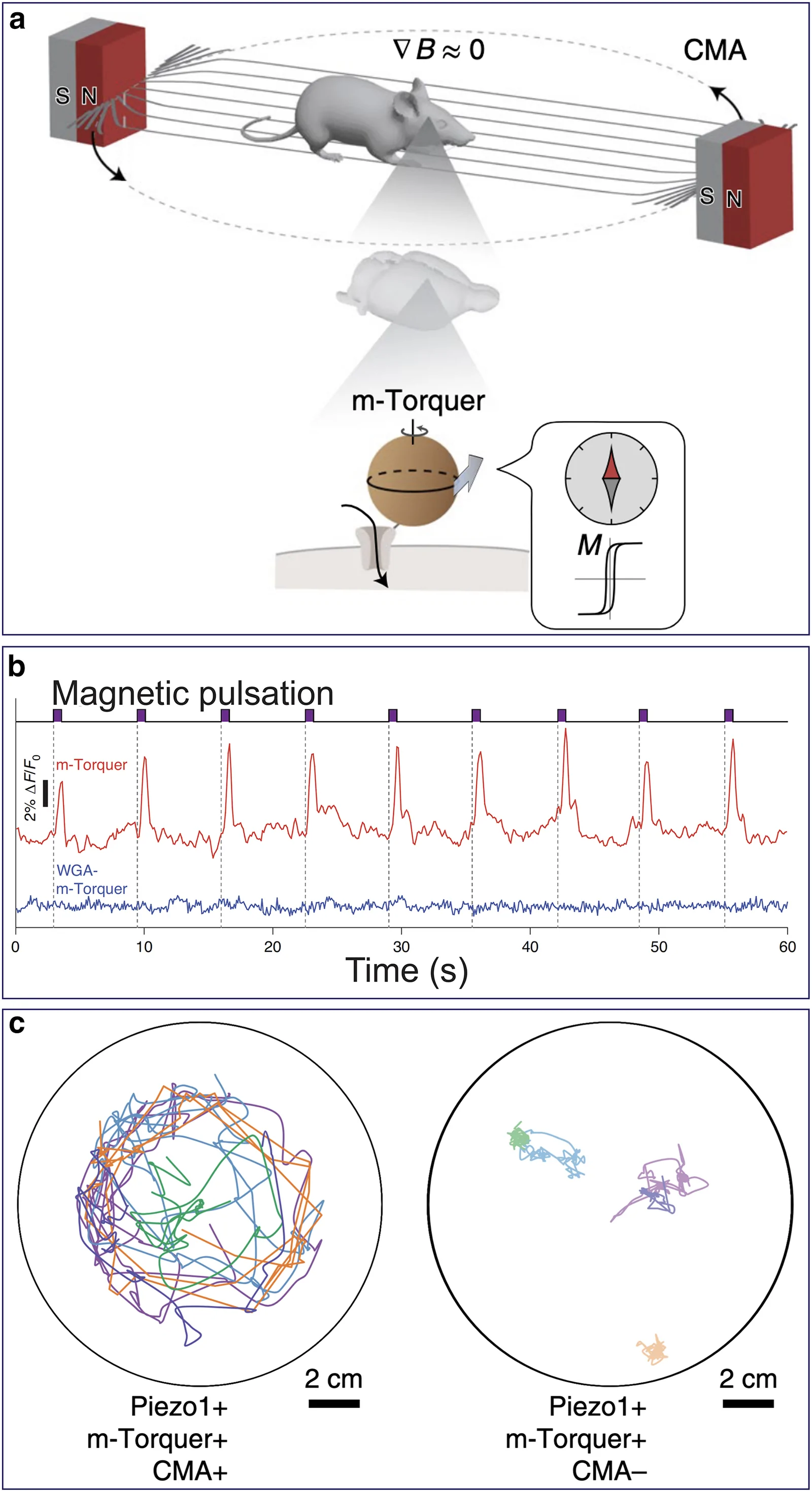
Nanoscale magnetic torquer (m-Torquer) for in vivo neuromodulation of mice. (*a*) The m-Torquer system combines a circular magnet array (CMA) producing a uniform rotating magnetic field (∇B ≈ 0) with a nanoparticle torque generator that acts as a nanoscale magnetic compass to deliver torque (Fτ) for magnetomechanical signaling. (*b*) Time-resolved Ca^2+^ influx measurements show repetitive stimulation of Piezo1 in neurons treated with m-Torquers (*red*) or WGA-m-Torquers (*blue*), using 0.5-s pulses at 0.5 Hz with 90° rotations. Results were consistent across three independent experiments. (*c*) Left: locomotion trajectories of five mice with m-Torquer in the right premotor cortex during CMA rotation (15 cycles, 2 s on/off). Right: control mice without CMA. Reprinted (adapted) with permission from *Nat. Mater.* 2021, 20, 1029–1036 (183). Copyright 2021, Springer Nature Limited.

Recently, the magnetic torque tweezer configuration has been modified to develop so-called m-Torquers, based on assemblies of nanoparticles, that can generate torque force to gate the ion channel activity of Piezo1 in mice (Fig. 4 *a*) (183). Compared with conventional MTs, which have been most effective to investigate single proteins at millimeter working distances (29), the separation of gradient and weak uniform fields in the m-Torquer-based design was reported to increase the working distance to ∼70 cm, enabling in vivo experiments in mice with external rotating magnetic fields. Thereby, repetitive activation of Piezo1 was achieved via torque force transduction (∼1.6 pN) in m-Torquer stimulated mice (Fig. 4 *b*). The applicability for neuromodulation by m-Torquers was tested on the locomotion of a mouse. Upon stimulation with a rotating circular magnet array, an enhancement in locomotion trajectories and speed was found compared with the control group (Fig. 4 *c*). Furthermore, MTs have also been applied to manipulate mitotic spindle orientation in vivo, enabling direct control of spindle positioning in developing sea urchin embryos (184). This approach allows quantification of the forces and torques required for spindle movement and provides insight into how spindle orientation influences developmental outcomes.

## Conclusion

MTs have firmly established themselves as a vital technique for probing protein mechanics under near-physiological conditions. Their ability to apply stable, subpiconewton forces over long timescales without photodamage, combined with high multiplexing and compatibility with fluorescence methods, has enabled unprecedented insight into force-dependent protein behavior. From uncovering the mechanical stability of cytoskeletal and membrane proteins to elucidating the role of mechanosensitive receptors like aGPCRs and ion channels, MTs have provided a powerful framework for linking molecular mechanics to biological function.

Despite this progress, several challenges and opportunities remain. One key challenge lies in expanding MT applicability to more complex and crowded cellular environments, where protein behavior is modulated by dynamic interactions with other biomolecules and membranes. Future efforts should aim to improve the integration of MTs with live-cell imaging and biochemical readouts to correlate force-dependent dynamics with functional outcomes in real time.

Moreover, achieving high spatiotemporal resolution in three-dimensional geometries such as within organoids or tissues will require novel engineering solutions, including more compact and mobile magnetic assemblies. Force calibration at subnanometer precision in nonlinear or torsionally constrained geometries remains a technical barrier. Addressing these issues will be crucial for advancing in vivo applications, such as probing force transduction during development, immune responses, or neuronal signaling.

On the molecular side, more systematic efforts are needed to map the mechanical energy landscapes of large, multidomain protein complexes and disordered protein regions. Although purely data-driven machine learning (ML) is often limited by data scarcity and interpretability in single-molecule experiments (185), approaches like AlphaFold have already demonstrated the transformative potential of ML for structural biology (186). Building on such successes, emerging hybrid strategies that combine ML with mechanistic modeling (e.g., physics-informed or state-discovery methods) may help reveal hidden intermediates and coupling terms, thereby offering richer insight into how forces shape folding pathways and signaling specificity (187,188).

In summary, MTs are uniquely positioned to lead the next phase of mechanobiology, offering a route to dissect the molecular logic by which mechanical forces are sensed, interpreted, and acted upon in living systems. Continued methodological innovation and interdisciplinary integration, along with the exploration of emerging force spectroscopy platforms, such as CFM and AFS, will be crucial to expand the scope of protein studies in mechanobiology and unlock their full potential for both fundamental discovery and biomedical applications.

## Data and code availability

This work is a review, and all data discussed have been previously published and are referenced in the article.

## Acknowledgments

We thank our colleagues in the force spectroscopy and biophysics community for many stimulating discussions. In particular, J.L. would like to thank Prof. Erich Sackmann for advice and insightful discussions while sharing an office at the LMU Munich.

This work was supported by the 10.13039/501100000781European Research Council through the Consolidator grant “ProForce” and the Alexander-von-Humboldt Foundation through a Feodor-Lynen fellowship.

## Author contributions

S.D.P.: conceptualization; literature collection; writing of initial draft; and editing, reviewing, and refinement of the manuscript. J.L.: conceptualization; literature collection; editing, reviewing, and refinement of the manuscript; supervision; and funding.

## Declaration of interests

The authors declare no competing interests.
